# Determinants of unsuccessful tuberculosis treatment outcomes among patients under facility-based directly observed therapy (DOT) follow-up in Rwanda (2021–2023): a retrospective analysis of national surveillance data

**DOI:** 10.1186/s12879-026-14148-y

**Published:** 2026-07-31

**Authors:** Kubwimana Richard, Ndagijimana Albert

**Affiliations:** 1 Department of Epidemiology and Biostatistics, School of Public Health, College of Medicine and Health Sciences, University of Rwanda, Kigali, Rwanda; 2 Gatonde District Hospital, Gakenke, Rwanda

**Keywords:** Tuberculosis, Directly observed therapy, HIV co-infection, Loss to follow-up, Death

## Abstract

**Background:**

Tuberculosis (TB) causes more than one million deaths annually. Achieving high treatment success is essential to interrupt transmission, yet unsuccessful treatment outcomes persist despite standardized facility-based directly observed therapy (DOT). This study assessed determinants of unsuccessful TB treatment among patients under facility-based directly observed therapy in Rwanda.

**Methods:**

We conducted a nationwide retrospective analysis of 9,149 TB patient records reported in Rwanda’s national electronic TB database (e-TB) covering July 2021–December 2023. We included drug-susceptible TB patients managed under standard treatment and facility-based DOT across 568 health facilities, including correctional institutions. The outcome was unsuccessful treatment, defined as death, treatment failure, or loss to follow-up. Multivariable logistic regression identified independent determinants, reported as adjusted odds ratios (aORs) with 95% confidence intervals (CIs).

**Results:**

Among 9,149 TB patients, 11% experienced unsuccessful treatment outcomes, primarily due to death (8%). In outcome-specific analysis, increasing age was associated with death (40–59 years: aOR 1.60, 95% CI 1.32–1.94; ≥60 years: aOR 2.80, 95% CI 2.24–3.49) but was inversely associated with loss to follow-up. HIV co-infection was independently associated with both death (aOR 2.99, 95% CI 2.47–3.61) and loss to follow-up (aOR 2.42, 95% CI 1.77–3.29). Compared with correctional facilities, treatment in hospitals showed an association with death (aOR 11.96, 95% CI 8.42–16.99), while both hospitals and health centres/private clinics were associated with increased loss to follow-up. Clinically diagnosed tuberculosis was associated with higher odds of death but lower odds of loss to follow-up. Marked geographic variation was observed, with higher mortality in the Northern and Southern provinces and increased loss to follow-up in Kigali City.

**Conclusions:**

The tuberculosis treatment success rate for patients receiving health facility-based DOT in Rwanda (89%) was slightly below the national average of 90% indicating room for improvement. Unsuccessful outcomes were mainly driven by death and were associated with older age, HIV co-infection, and health system factors, particularly treatment in hospitals and health centres compared with correctional facilities. To reduce unsuccessful treatment outcomes, programs should prioritize continuity of care and differentiated support for high-risk groups, particularly older adults and HIV-positive patients.

**Supplementary Information:**

The online version contains supplementary material available at https://doi.org/10.1186/s12879-026-14148-y.

## Background

More than 1 million people die of tuberculosis every year, ranking it among the top ten causes of death in the world [1]. The fatality of tuberculosis is also shown by the fact that it is the leading cause of death attributable to a single infectious agent [2, 3]. It is estimated that approximately a quarter of the world’s population has experienced TB infection [4]. Globally, 10.7 million people fell ill with tuberculosis and an estimated 1.23 million lives were lost in 2024. Although progress has been substantial, Africa still shares 25% of the global TB burden [1].

The World Health Organisation (WHO) and all Member States of the United Nations have set a bold vision of ending the tuberculosis epidemic by 2035 [5]. To achieve this, it was recommended to measure the performance of TB control programs based on treatment outcomes. Five distinct categories were established for evaluating TB treatment: cured, treatment completed, treatment failed, died, and lost to follow-up. Successful treatment outcomes were defined as cases that are either cured or have completed treatment, while unsuccessful treatment outcomes include cases where treatment failed, patients who were lost to follow-up, or patients died [6, 7]. In 2024, the global treatment success rate for drug-susceptible tuberculosis (DS-TB) was 88% [1]. DS-TB is a bacteriologically confirmed or clinically diagnosed case without strains resistant to rifampicin and isoniazid [3].

Tuberculosis (TB) remains a major public health challenge in Rwanda, a country with an estimated population of approximately 13 million. According to World Health Organization (WHO) estimates, the national TB incidence ranges between 55 and 99 cases per 100,000 population [1]. In 2024, the country reported 8,551 TB cases underscoring the burden of disease, this indicates persistent transmission despite ongoing control efforts. In 2024, Rwanda achieved an overall treatment success rate of 90% for drug-susceptible tuberculosis but unsuccessful treatment outcomes persist. Death, which was the leading adverse outcome has claimed 520 lives, accounting for 5.6% (520/9,351) of all susceptible TB cases in 2024 alone [8].

TB treatment typically lasts at least six months, making adherence challenging. To address this, directly observed therapy (DOT) was introduced in the 1950s as a pilot approach to ensure treatment completion. In 1994, the World Health Organisation formally adopted DOT as a cornerstone of global TB control, following evidence of its effectiveness in improving adherence and preventing drug resistance [9–11]. In practice, DOT involves a healthcare worker, community health worker, or family member observing patients as they take their medication, fostering collaboration between patients and the health system [12, 13].

Directly Observed Treatment (DOT), particularly in community-based models, has been consistently associated with improved tuberculosis treatment outcomes across diverse settings. Evidence from Brazil showed a 25% reduction in unsuccessful treatment outcomes following DOT implementation, highlighting its contribution to improved adherence and treatment continuity [14]. In Ethiopia, the introduction of a community-based TB care package substantially increased treatment success rates from 76% to 95%, reflecting the effectiveness of decentralised, patient-centred care models in improving TB outcomes [10, 15]. Similar improvements have also been reported in Thailand, where strengthened community-based support mechanisms enhanced treatment completion rates. A systematic review published in PLOS One further synthesised global evidence and concluded that community-based DOT has been associated with improved TB treatment outcomes across settings, primarily through improved adherence, reduced loss to follow-up, and strengthened patient supervision [16].

Evidence suggests that community-based DOT, when delivered by trained and supervised community healthcare workers, is associated with high TB treatment success [9, 17, 18]. Health facility-based DOT treatment success has been documented with significant variability. This variability may be explained by patient travel costs, opportunity costs of clinic visits, and stigma associated with daily attendance at health facilities—factors that are less prominent in community-based DOT models. For health facility-based DOT, success rates have been recorded as low as 21% and as high as 92.3% [19]. Unsuccessful treatment outcomes have been primarily attributed to missed opportunities and long waiting time and distances required to access medication at health facilities [20, 21]. A systematic search of PubMed and African Journals Online identified no peer-reviewed study examining determinants of facility-based DOT outcomes within Rwandan context. This study aimed to determine factors independently associated with unsuccessful TB treatment outcomes among patients receiving facility-based DOT in Rwanda.

## Methods

### Study design

We conducted a retrospective analysis of national surveillance data from Rwanda’s electronic tuberculosis reporting system (e-TB), covering July 2021 to December 2023. The directly observed therapy modality variable was only routinely collected from July 2021 onward justifying the choice of study period.

### Study setting

This nationwide study was conducted in Rwanda, a landlocked East African country divided into four provinces and Kigali City, comprising 30 districts. The health system mirrors the administrative structure: community health workers at the village level provide basic care; health centres at the sector level and district hospitals deliver treatment; and correctional facilities also contribute to TB care, with DOT implemented across all levels to ensure adherence. In total, 571 facilities (hospitals, health centres, and correctional facilities) report TB data through the e-TB system, where each anti-TB dose is observed by a healthcare provider.

### Study population

The study included patients diagnosed with drug-susceptible tuberculosis (DS-TB) who were registered in the national e-TB database and managed under documented facility-based DOT follow-up between July 2021 to December 2023. All included patients received standard anti-TB treatment according to national tuberculosis treatment guidelines in Rwanda. Due to differences in treatment regimens, outcomes and follow-up definitions, we excluded patients with multidrug-resistant tuberculosis (MDR-TB). We also excluded records with missing or incomplete key variables, including undocumented treatment outcomes or missing DOT modality information.

### Sampling

**Fig. 1:**
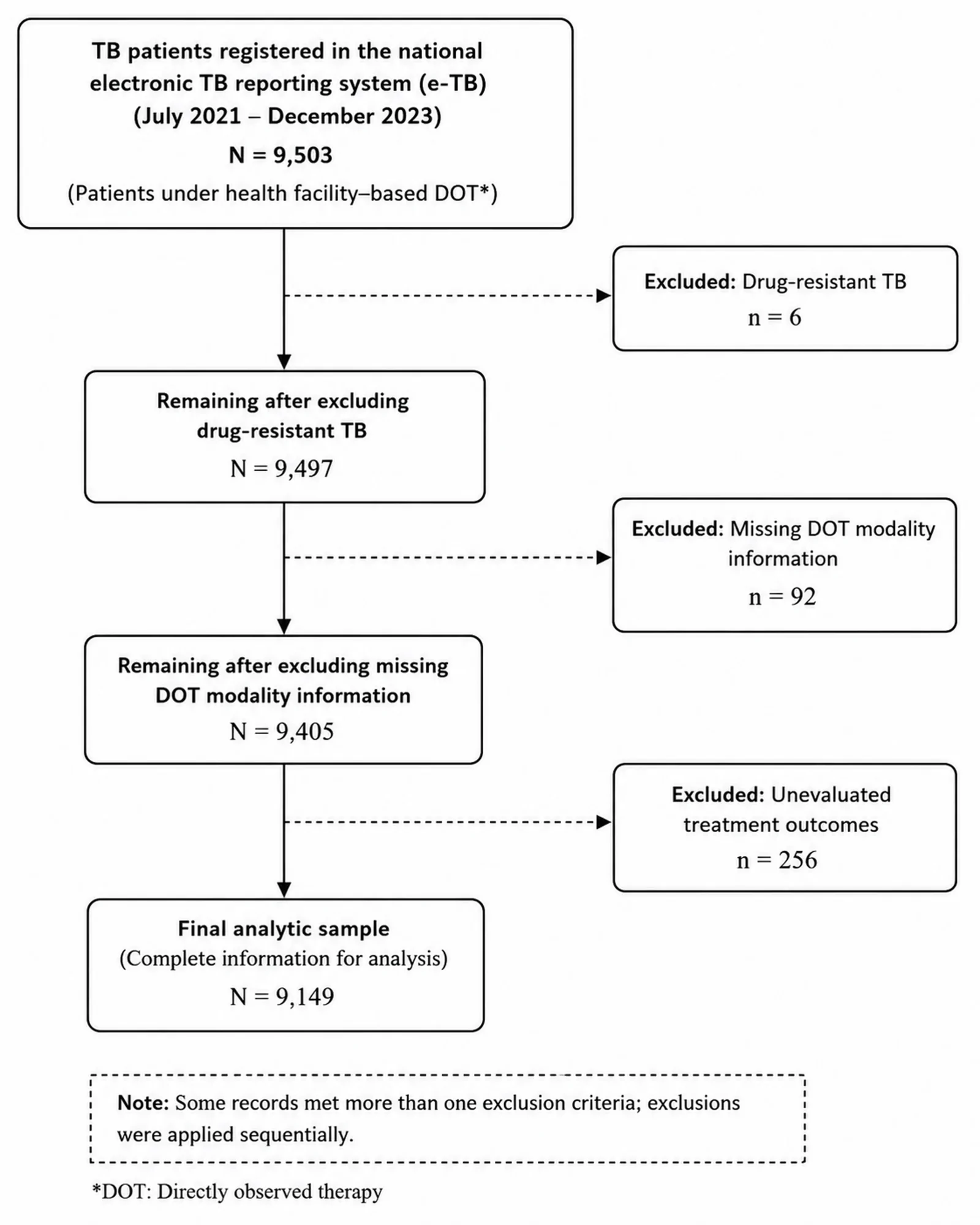
Flow diagram of selecting study participants

This study included all eligible patients meeting the inclusion criteria. A total of 9,503 tuberculosis (TB) patients managed under facility-based directly observed therapy (DOT) were reported from the national electronic TB reporting system (e-TB) between July 2021 and December 2023. Patients with drug-resistant TB (*n* = 6), missing DOT modality information (92), unevaluated treatment outcomes (*n* = 256) were excluded. After data cleaning and application of eligibility criteria, the final analytic sample comprised 9,149 patients with complete information for analysis (Fig. 1. Flow diagram of patient selection).

### Data extraction methods

Patient information is collected at tuberculosis (TB) clinics and recorded in individual patient files during the diagnostic process. Data managers subsequently enter these records into the electronic TB reporting system (e-TB). At national level, quarterly data quality assessments are conducted in each district, during which patient files are reviewed and cross-checked against e-TB records to identify and resolve discrepancies in data accuracy and completeness. This standardized data collection and verification system is implemented uniformly across all districts in Rwanda. For this study, TB patient records were extracted from the national e-TB database as an analytical dataset for further processing and statistical analysis.

### Study variables

In this study, the outcome variable was TB treatment outcome. Tuberculosis treatment outcomes were classified as either successful or unsuccessful. Successful outcomes included patients who were either *cured*—demonstrating negative smear or culture results in the final month of treatment and at least once earlier—or who *completed treatment* without bacteriological confirmation of cure. Unsuccessful treatment outcomes comprised *treatment failure* (persistent positive smear or culture at the fifth month or later), *loss to follow-up* (patients who missed treatment for two or more consecutive months), and *death* from any cause before or during the course of treatment.

As independent variables, we had the patient’s age in years categorised into three categories (< 40, 40–59 and 60+). Residence recorded in provinces/city such as Kigali City, Eastern, Western, Northern, and Southern provinces. Sex was recorded as male and female. Type of treating health facility recorded as Hospitals, Correctional Facilities (Prisons) or Health Centres/Private Clinics. Health facility management is recorded as Public or Faith-based & Private. The level of Care provided by the entity is recorded as CDT (Diagnosis and Treatment Centre) or CT (Treatment Centre). HIV status was dichotomized into positive or negative.

Use of cotrimoxazole was categorized as “Yes” for HIV-positive patients receiving cotrimoxazole, “No” for HIV-positive patients not taking cotrimoxazole. BMI at the initiation of TB treatment was categorized as BMI less than 18.5 kg/m² and BMI equal to or greater than 18.5 kg/m². TB contact history was dichotomized as “Yes” or “No.” Receipt of TB nutritional support was dichotomized as “Yes” or “No.” The method of TB confirmation was classified as clinically diagnosed tuberculosis or bacteriologically confirmed. The Site of TB disease was dichotomized as pulmonary or extrapulmonary tuberculosis.

### Data analysis

The extracted data were exported to Stata version 17.0 (Stata Corp LLC) for cleaning and analysis. Descriptive statistics were plotted to summarize participants’ demographic and clinical characteristics, using means and standard deviations, medians and ranges for continuous variables as appropriate, and frequencies and percentages for categorical variables. Bivariate analysis was performed using binary logistic regression to determine the relationship between each independent variable and the dependent variable. Variables statistically significant in bivariate analysis were included in the multivariable logistic regression model to determine the independent factors associated with unsuccessful TB treatment outcomes. Multicollinearity was assessed using variance inflation factors (VIF); all VIF values were < 2.5, indicating no significant collinearity. To account for potential correlation of outcomes within the same health facility, we also fitted a mixed-effects logistic regression model with random intercepts for facility. Results were similar to the standard logistic regression suggesting minimal clustering effect. We reported adjusted odds ratios (aORs) and 95% confidence intervals (CIs).

## Results

### Description of study participants

This study analysed data from 9,149 tuberculosis patients, with a mean age of 40 years (SD = 16). Over half of the patients (55%) were younger than 40 years, and males accounted for the majority (80%), consistent with known gender disparities in TB burden. Geographically, the Eastern Province and Kigali City reported the highest proportions of cases, at 37% and 33% respectively, while the Northern Province contributed only 6%. The majority of patients received care in hospitals (42%) or correctional facilities (35%), with public health facilities accounting for 89% of cases (Table 1).

**Table 1:** Socio-demographic and Healthcare-related Characteristics of study participants (*N* = 9,149)

Variable	Frequency (*n*)	Percentage (%)
**Age group (years)**		
Mean (SD)*		40 (16)
< 40	5,066	55
40–59	2,933	32
≥ 60	1,150	13
**Sex** ^**a**^		
Male	7,275	79
Female	1,871	20
**Province of residence**		
East	3,392	37
Kigali	3,056	33
South	1,261	14
West	942	10
North	498	6
**Type of treating health facility**		
Hospitals	3,854	42
Correctional facilities (Prisons)	3,209	35
Health centres/Private clinics	2,086	23
**Health Facility Management**		
Public	8,102	89
Faith-based & Private	1,047	11
**Level of Care**		
CDT†	8,297	91
CT‡	852	9

^a^Three participants had missing sex information

* Mean age presented as mean (standard deviation)

† CDT = Centre de Diagnostic et de Traitement

‡ CT = Centre de Traitement

### Clinical characteristics of study participants

HIV co-infection was observed in 14% of patients, while among HIV-positive individuals, 52% were on cotrimoxazole prophylaxis. Cotrimoxazole information was available only among HIV-positive patients with documented prophylaxis records. TB contact history was reported in 16% of cases.

Most (88%) patients had a BMI < 18.5 kg/m^2^ at treatment initiation, indicating widespread undernutrition. Despite this, only 23% received TB nutritional support. Pulmonary TB was the most common form (75%). TB diagnosis was predominantly bacteriological (Table 2).

**Table 2:** Clinical characteristics of tuberculosis patients under facility-based DOT follow-up in Rwanda, July 2021–December 2023 (*N* = 9,149)

Variable	Frequency (*n*)	Percentage (%)
**HIV Status**		
Negative	7857	86
Positive	1288	14
**Currently on cotrimoxazole** ^**a**^		
Yes	663	52
No	610	48
**TB contact history**		
Yes	1501	16
No	7648	84
**BMI at treatment initiation**		
< 18.5 kg/m²	8010	88
≥ 18.5 kg/m²	1139	12
**Receipt of TB nutritional support**		
Yes	2103	23
No	7046	77
**Site of TB disease**		
Pulmonary TB	6827	75
Extra-pulmonary TB	2322	25
**Method of TB confirmation**		
Clinically diagnosed TB	3272	36
Bacteriologically confirmed	5877	64

^a^Cotrimoxazole analysis restricted to HIV-positive patients

### Treatment outcome for study participants

This study found an overall treatment success rate of 89%, 979 (11%) of patients experienced unsuccessful treatment outcomes, with mortality emerging as the most significant contributor (Table 3).

**Table 3:** Treatment Outcomes of study participants (*N* = 9,149)

TB Treatment Outcome	Frequency (*n*)	Percentage (%)	
**Successful treatment**	**8170**	**89**	
Cured	4649	51	
Completed	3521	38	
**Unsuccessful treatment**	**979**	**11**	
Died	697	8	
Lost to follow-up	229	2	
Failure	53	1	
**Total**	**9149**	**100**	

### Factors associated with unsuccessful TB treatment

In bivariate analysis, older age was significantly associated with unsuccessful treatment outcomes, with patients aged 60 + having 2.4 times higher odds of unsuccessful treatment (cOR = 2.36, CI = 1.97–2.84) compared to those under 40. Female patients had 1.6 times the odds (cOR = 1.60, CI = 1.38–1.86). However, this association was not significant after multivariable adjustment (aOR = 0.80, CI = 0.72-1.00). Patients from the Northern province had approximately twice the odds of unsuccessful treatment (cOR = 2.04, CI = 1.49–2.79) compared to those from the Western province. Patients treated in hospitals (cOR: 9.7, CI: 7.5–12.4) and health centres (OR: 5.0, CI: 3.8–6.6) had significantly higher odds of unsuccessful treatment outcomes than those being treated in correctional facilities. TB-HIV coinfected patients had 3.4 times higher odds of unsuccessful treatment ( cOR = 3.45, CI = 2.97–4.01). Patients without prior TB contact history had 4.39 times the odds of unsuccessful treatment ( cOR: 4.39, CI = 3.25–5.94) than those who were not. Clinically diagnosed TB cases had nearly 40% higher odds of unsuccessful treatment outcomes than bacteriologically confirmed cases (cOR = 1.37, CI = 1.20–1.57) (Supplementary File 1).

In multivariable logistic regression analysis, six factors were independently associated with increased odds of unsuccessful treatment outcomes. Compared with patients aged < 40 years, those aged 40–59 years had higher odds of unsuccessful treatment outcomes (adjusted odds ratio [aOR] 1.21; 95% CI: 1.03–1.41), while patients aged ≥ 60 years had nearly twice the odds (aOR 1.98; 95% CI: 1.63–2.40).

Using Western Province as reference, significantly higher odds of unsuccessful treatment outcomes were observed among patients residing in Eastern Province (aOR 1.98; 95% CI: 1.49–2.62), Kigali City (aOR 1.49; 95% CI: 1.16–1.90), Northern Province (aOR 2.13; 95% CI: 1.54–2.94), and Southern Province (aOR 2.33; 95% CI: 1.76–3.09). Compared with correctional facilities (prisons), treatment in health centres or private clinics was associated with higher odds of unsuccessful treatment outcomes (aOR 4.63; 95% CI: 3.36–6.36), while treatment in hospitals showed even greater odds (aOR 9.11; 95% CI: 6.79–12.21).

To account for potential correlation of outcomes within health facilities, a mixed-effects logistic regression model with random intercepts for facility was fitted as a sensitivity analysis. Results were consistent with the standard logistic regression model indicating minimal clustering effect.

Among clinical factors, HIV-positive status was significantly associated with unsuccessful treatment outcomes (aOR 2.69; 95% CI: 2.29–3.16). Absence of known contact with an index tuberculosis case was also associated with higher odds of unsuccessful treatment outcomes (aOR 1.66; 95% CI: 1.20–2.29) (Table 4).

**Table 4:** Multivariable analysis of factors associated with unsuccessful TB treatment outcomes for TB patients under health facility-based DOT in Rwanda

Variable	Adjusted OR (95% CI)
**Age groups (Years)**	
< 40	Reference
40–59	**1.21** (1.03–1.41)
60+	**1.98** (1.63–2.40)
**Sex**	
Female	0.8 ( 0.72-1.0)
Male	Reference
**Province of residence**	
West	Reference
East	**1.98** (1.49–2.62)
Kigali	**1.49** (1.16–1.90)
North	**2.13** (1.54–2.94)
South	**2.33** (1.76–3.09)
**Type of treating health facility**	
Correctional Facilities (Prisons)	Reference
Health Centers/Private Clinics	**4.63** (3.36–6.36)
Hospitals	**9.11** (6.79–12.21)
**HIV status**	
Negative	Reference
Positive	**2.69** (2.29–3.16)
**TB contact history**	
Yes	Reference
No	**1.66** (1.20–2.29)
**Method of TB confirmation**	
Bacteriologically confirmed	Reference
Clinically diagnosed	1.16 (1.00–1.36)
**Level of Care**	
CT	Reference
CDT	1.30(0.96–1.77)

*DOT: Directly-observed therapy

^+^Western Province was used as the reference category because it demonstrated the lowest crude proportion of unsuccessful treatment outcomes; however, Southern Province showed the highest adjusted odds in multivariable analysis

### Subgroup analysis by type of unsuccessful outcome

Among 979 unsuccessful treatment outcomes, 697 (71%) were deaths, 229 (23%) were loss to follow-up, and 53 (5%) were treatment failure. In multivariable analyses of each type of unsuccessful outcome namely death and loss to follow-up (failure was rare, *n* = 53) for reliable multivariable modelling), older age was an associated factor of mortality, with patients aged 40–59 years and ≥ 60 years having 1.60-fold (95% CI 1.32–1.94) and 2.80-fold (95% CI 2.24–3.49) higher odds of death, respectively, compared with those under 40 years; conversely, older age was protective against loss to follow-up (LTFU). HIV co-infection was independently associated with both death (aOR 2.99, 95% CI 2.47–3.61) and LTFU (aOR 2.42, 95% CI 1.77–3.29). Clinically diagnosed TB, compared with bacteriologically confirmed cases, was associated with higher odds of death (aOR 1.48, 95% CI 1.24–1.77) but lower odds of LTFU (aOR 0.69, 95% CI 0.48–0.98). Geographic variation was evident, with patients in the Southern, Northern, and Eastern provinces at higher risk of death, while Kigali City and Eastern and Southern provinces had the highest odds of LTFU, relative to Western Province. The type of treating facility was associated with mortality: compared with correctional facilities, patients managed at hospitals had nearly 12-fold higher odds of death (aOR 11.96, 95% CI 8.42–16.99). Sex and TB contact history were not significantly associated with either outcome in multivariable analysis (Table 5).

**Table 5:** Multivariable factors associated with death and loss to follow-up among tuberculosis patients compared with successful treatment outcomes (Death, loss to follow-up)

Variable	Death (*n* = 697) aOR (95% CI)	Loss to follow-up (*n* = 229) aOR (95% CI)
**Age group (years)**		
< 40	Reference	Reference
40–59	**1.60** (1.32–1.94)	**0.55** (0.40–0.75)
≥ 60	**2.80** (2.24–3.49)	**0.40** (0.22–0.72)
**Sex**		
Male	Reference	Reference
Female	0.85 (0.71–1.02)	0.88 (0.60–1.08)
**Province of residence**		
Western	Reference	Reference
East	**2.02** (1.47–2.77)	**2.65** (1.21–5.79)
Kigali City	1.17 (0.88–1.55)	**4.17** (2.10–8.27)
North	**2.23** (1.56–3.19)	2.24 (0.91–5.47)
South	**2.48** (1.81–3.38)	**2.84** (1.30–6.19)
**Type of treating health facility**		
Correctional facilities (Prisons)	Reference	Reference
Health centre / Private clinic	**3.86** (2.46–6.07)	**4.86** (2.54–9.30)
Hospital	**11.96** (8.42–16.99)	**3.42** (1.87–6.25)
**HIV status**		
Negative	Reference	Reference
HIV positive	**2.99** (2.47–3.61)	**2.42** (1.77–3.29)
**Method of TB confirmation**		
Bacteriologically confirmed	Reference	Reference
Clinically diagnosed TB	**1.48** (1.24–1.77)	**0.69** (0.48–0.98)
**TB contact history**		
No	Reference	Reference
Yes	1.46 (0.99–2.15)	1.82 (0.97–3.40)

*aOR = adjusted odds ratio; CI = confidence interval

Estimates obtained from separate multivariable logistic regression models comparing death, loss to follow-up with successful treatment outcomes

## Discussion

### Treatment success rate

This study assessed rates and determinants of unsuccessful TB treatment outcomes among patients on facility-based DOT in Rwanda. The findings indicate that 11% of TB patients experienced unsuccessful treatment outcomes corresponding to a treatment success rate of 89%. The global treatment success rate for drug-susceptible tuberculosis was approximately 88% [1]. Although there might be structural differences in healthcare systems, DOT type, HIV burden compared with other settings, Rwanda’s treatment outcomes were more favourable than those reported in Brazil, Nigeria and Kenya, where 21%, 24% and 22.8% of tuberculosis patients experienced unsuccessful outcomes, respectively [22–24]. Rwanda’s strong performance may be attributed to the effective implementation of the directly observed therapy (DOT), integrated TB-HIV services, and a strong programmatic supervision. In addition, regular monitoring by the Rwanda Biomedical Centre (RBC) and district-level supervision likely improved treatment adherence and follow-up.

### Factors associated with unsuccessful treatment outcomes

We found that in this study, older patients, TB patients residing in the northern and southern provinces, TB-HIV co-infection, receiving care at hospitals, and health centres, had higher odds of unsuccessful treatment outcomes. Consistent with expectations, documented contact with an index TB case was associated with lower odds of unsuccessful treatment, likely due to earlier diagnosis and less advanced disease. However this may be subject to recall or reporting bias and may reflect health-seeking behaviour rather than biological protection.

Increasing age was associated with increased odds of unsuccessful TB treatment outcomes. Unsuccessful treatment outcomes in older patients are likely due to immunological decline and comorbidities, and the challenges in completing TB treatment, which is often long-duration [25, 26]. Older patients also face higher risks of pulmonary tuberculosis, which is known to be linked with higher mortality, as shown by a study done at King Abdulaziz Medical City in Riyadh [27].

Regarding place of residence, patients residing in the Eastern, Northern, and Southern Provinces, as well as Kigali City, showed higher odds of unsuccessful treatment outcomes compared to those in the Western Province. This regional variation is consistent with a study conducted in Malawi, where geographical disparities in health system performance, access to diagnostics, and follow-up level influenced TB outcomes [28]. Notably, a study in Zambia emphasised that urban areas, despite having more facilities, often face higher patient loads and fragmented care, which may explain the increased risk seen in Kigali City [29]. These findings underscore the importance of strengthening localised TB control strategies and addressing structural inequalities that hinder treatment success. The change in direction between crude and adjusted estimates for Eastern Province suggests confounding, likely related to the high concentration of correctional facilities within the province.

The type of health facility was a factor associated with unsuccessful treatment outcomes, with patients treated in hospitals and health centres/private clinics showing higher odds of unsuccessful treatment compared with those managed in correctional facilities. This finding may reflect differences in care delivery models, where correctional settings provide more structured treatment supervision and closer adherence monitoring, which has been shown to improve treatment continuity and outcomes [26, 30]. In contrast, hospital- and health centre-based care may be affected by longer travel distances, higher patient volumes, and less intensive follow-up mechanisms, all of which can disrupt continuity of care and contribute to treatment interruption and poor outcomes [23, 31]. However, these associations should be interpreted cautiously due to likely residual confounding as population characteristics may differ. Patients treated in hospitals are often older and clinically more complex, with higher comorbidity burden and more advanced disease at presentation. Although the analysis adjusted for age and HIV status, unmeasured factors such as baseline disease severity, delayed diagnosis, and clinical instability may partly explain the observed associations. Strengthening linkage systems between hospitals, health centres, and community-based follow-up structures may therefore improve continuity of care and reduce mortality and loss to follow-up among high-risk tuberculosis patients.

HIV co-infection was independently associated with poorer TB outcomes, consistent with global evidence linking HIV to delayed sputum conversion, increased risk of relapse, and higher mortality. A study done in Accra Regional Hospital of Ghana found that there was a 13% TB treatment success rate difference when there was TB-HIV coinfection than when there was only TB. HIV co-infection increased the odds of unsuccessful treatment outcomes by approximately threefold [32]. The same findings were reported in Ethiopia at Adare Hospital, where a TB patient was eight times more likely to die if he had HIV [33].

Despite 88% of patients being underweight (BMI < 18.5 kg/m^2^) at treatment initiation, provision of TB nutritional support (23% of patients) was not associated with treatment outcomes in bivariate analysis (cOR = 1.06; 95% CI 0.90–1.24). This likely reflects the advanced nutritional vulnerability among patients initiating TB treatment. The absence of association may also reflect confounding by indication, whereby nutritional support is preferentially provided to patients with more severe malnutrition or advanced disease rather than lack of intervention effectiveness [34]. Nevertheless, nutritional assessment and support remain recommended components of tuberculosis care according to WHO guidance [35]. Prospective studies using standardized nutritional interventions are needed to better evaluate causal effects.

Patients with a documented history of contact with an index TB case had 40% lower odds of experiencing unsuccessful treatment outcomes compared to those without such contact. This protective association although it needs further investigation, is probably attributed to earlier case detection, prompt initiation of treatment [6, 36].

### Subgroup analysis by type of unsuccessful treatment outcome

The subgroup analysis demonstrated that factors associated with unsuccessful tuberculosis (TB) treatment outcomes differed between death and loss to follow-up, indicating that these outcomes represent distinct clinical and programmatic challenges rather than a single homogeneous endpoint. Increasing age was strongly associated with mortality. This pattern is consistent with a scoping review that found that higher TB mortality among older adults may largely be attributable to comorbidities, delayed diagnosis, and difficulties in symptom recognition and timely healthcare seeking [25]. In contrast to several previous studies where older age was identified as a factor for loss to follow-up during TB treatment, there was an inverse association of age with loss to follow-up. Tuberculosis patients aged above 40 years had lower odds of loss to follow-up compared with younger patients [37]. Although this needs further investigation, particularly through prospective and qualitative studies exploring age-specific barriers to TB treatment adherence, a possible explanation is that younger adults may experience greater mobility, employment-related migration, competing socioeconomic responsibilities, and lower perceived disease severity, all of which could negatively affect treatment continuity. These contrasting age-related patterns suggest that mortality and loss to follow-up are driven by different underlying risk profiles and may therefore require distinct intervention strategies.

HIV co-infection remained independently associated with both death and loss to follow-up. Similar findings have been reported in Brazil and other high HIV-burden settings, where immunosuppression, pill burden, drug interactions, and complex care pathways adversely affect TB treatment outcomes [38]. However, our findings differ from a study conducted in northern France, where TB/HIV co-infected patients had lower odds of loss to follow-up, possibly because frequent HIV-related clinic visits enhanced continuity of care and treatment adherence [39]. The persistent association observed in this study reinforces WHO recommendations advocating for integrated TB/HIV services to improve treatment retention and survival outcomes.

Differences were also observed according to diagnostic classification. Clinically diagnosed TB was associated with increased mortality compared with bacteriologically confirmed disease. Similar findings have been reported elsewhere, where clinically diagnosed patients frequently present with advanced disease, extrapulmonary TB, or severe clinical deterioration requiring empirical treatment initiation before microbiological confirmation [40, 41]. These conditions are often associated with delayed diagnosis and poorer prognosis. Conversely, the lower odds of loss to follow-up among clinically diagnosed patients may reflect closer clinical monitoring and intensified follow-up once severe disease is suspected.

Geographic variation in both mortality and loss to follow-up was evident across provinces. Similar regional heterogeneity has been described in national TB programme evaluations, where disparities in healthcare accessibility, urbanization, socioeconomic conditions, and health system performance contribute to unequal treatment outcomes [42]. However, these geographical differences may also reflect unmeasured socioeconomic and health system factors not captured in the present study. The higher loss to follow-up observed in Kigali City is consistent with findings from a registry-based study conducted in an industrial state of Malaysia, where urban TB patients were more likely to interrupt treatment because of population mobility and fragmented continuity of care [43].These findings suggest that urbanization alone does not necessarily improve treatment adherence and may introduce additional barriers to continuity of care.

Health facility level also influenced treatment outcomes. Patients managed at hospitals and health centres/private clinics had higher odds of both mortality and loss to follow-up compared with those treated in correctional facilities. This association may partly reflect referral of older and more severely ill patients to higher-level facilities, as well as differences in treatment supervision and continuity of care [26]. In contrast, correctional facilities typically operate within highly structured environments where directly observed therapy and patient monitoring can be implemented more consistently with no travel barriers, mandatory attendance and structured schedule. Similar findings have been reported in prison-based TB studies demonstrating higher treatment completion rates associated with structured supervision systems [30]. The present findings therefore support existing evidence suggesting that treatment adherence may be strengthened in controlled treatment environments, whereas routine healthcare settings may face greater challenges in sustaining long-term patient retention [44].

Overall, the differing factors associated with death and loss to follow-up highlight the importance of differentiated TB control strategies. Interventions aimed at reducing mortality should prioritize early identification and intensified clinical management of older adults and HIV co-infected patients. In contrast, strategies to reduce loss to follow-up should emphasize patient-centred adherence support, continuity of care, and retention mechanisms targeting younger and urban populations. Strengthening integration between hospitals, health centres, and community follow-up systems may further improve continuity of care among high-risk patients.

### Study strengths and limitations

The nationwide scope and large sample size provide a comprehensive overview of TB treatment outcomes across Rwandan health facility-based DOT, thereby increasing the generalizability of the results. However, there are some limitations that should be acknowledged. First, this study relied on secondary surveillance data from the national electronic TB reporting system (e-TB), which is subject to missing or incompletely recorded variables. Important clinical, socioeconomic, and behavioural determinants of TB outcomes—including treatment adherence, nutritional status, income, education level, substance use, and drug supply interruptions—were not available and therefore could not be assessed. Additionally, the e-TB system does not capture drug stock-outs or side effect profiles, both of which are known drivers of treatment failure and loss to follow-up.

Finally, although analyses adjusted for facility characteristics, clustering of outcomes within individual facilities was not fully modelled in the primary analysis. Sensitivity analyses using mixed-effects models yielded similar results, suggesting minimal impact on overall conclusions. Because of the retrospective observational design, causal relationships between factors and TB treatment outcomes cannot be definitively established.

Despite these limitations, the study provides important national evidence on determinants of TB treatment outcomes within routinely implemented health facility–based DOT services in Rwanda and identifies areas for improving TB treatment success.

## Conclusion

Approximately one in nine patients receiving facility-based directly observed therapy (DOT) in Rwanda experienced unsuccessful tuberculosis treatment outcomes, predominantly death. Older age, HIV co-infection, provincial disparities, and treatment at hospitals or health centres/private clinics were independently associated with poorer outcomes. Given the nearly 2.7-fold higher odds of unsuccessful outcomes among HIV-positive patients, integrated TB-HIV services should be prioritised, particularly in provinces with high HIV burden. Strengthening patient-centred and decentralised TB care, improving continuity of follow-up, and enhancing contact tracing may further improve treatment success. Further prospective studies are needed to evaluate additional clinical and socioeconomic determinants not studied in this research.

## Supplementary Information

Below is the link to the electronic supplementary material.

Supplementary Material 1

## Data Availability

The datasets used (analysed) during the current study are available from the corresponding author on reasonable request.

## Abbreviations

aOR: adjusted Odds Ratio
BMI: Body Mass Index
CDT: Diagnosis and Treatment Centre
CI: Confidence Interval
cOR: Crude Odds Ratio
CT: Treatment Centre
DOT: Directly Observed Therapy
DS-TB: Drug-Susceptible Tuberculosis
e-TB: electronic Tuberculosis Reporting System
HIV: Human Immunodeficiency Virus
MDR-TB: Multidrug-Resistant Tuberculosis
OR: Odds Ratio
RBC: Rwanda Biomedical Centre
SD: Standard Deviation
TB: Tuberculosis
WHO: World Health Organization

## References

[1] World Health Organization. Global tuberculosis report 2025. Geneva: World Health Organization. 2025. Available from: https://www.who.int/publications/i/item/9789240116924. Accessed on 24 May 2026.

[2] Chhabra S, Ong CWM, Sotgiu G. TB diagnosis and treatment during the COVID-19 pandemic. Int J Tuberc Lung Dis. 2022;26(8):702–703. 10.5588/ijtld.22.0314. PMID: 35898138. 35898138

[3] World Health Organization. Global tuberculosis report 2023. Geneva: World Health Organization. 2023 Available from: https://www.who.int/publications/i/item/9789240083851 Accessed on 24 May 2026.

[4] World Health Organization. Global tuberculosis report 2022. Geneva: World Health Organization. 2022. Available from: https://www.who.int/publications/i/item/9789240061729 Accessed on 24 May 2026.

[5] World Health Assembly. Global strategy and targets for tuberculosis prevention, care and control after 2015 (Resolution WHA67.1, Agenda item 12.1). Available from: https://iris.who.int/handle/10665/162760 Accessed on 24 May 2026.

[6] Rwanda Ministry of Health. National guidelines for the management of tuberculosis. 2nd ed. Kigali, Rwanda: Institute of HIV/AIDS, Disease Prevention & Control (IHDPC), Tuberculosis & Other Respiratory Communicable Diseases Division;; 2020.

[7] Muluye AB, Kebamo S, Teklie T, Alemkere G. Poor treatment outcomes and its determinants among tuberculosis patients in selected health facilities in East Wollega, Western Ethiopia. PLoS ONE. 2018;13(10):e0206227. 10.1371/journal.pone.0206227. PMID: 30365530; PMCID: PMC6203372. PMC6203372 30365530

[8] Rwanda biomedical centre (Ministry of Health, Rwanda). National tuberculosis and other respiratory communicable diseases program annual report 2023–2024, Kigali. Rwanda: Rwanda biomedical centre; 2024. Available from: https://rbc.gov.rw/fileadmin/user_upload/report_2024/TB/TBORD_annual_report_2023-2024.pdf. Accessed on 24 May 2026.

[9] Prasad BM, Chadha SS, Thekkur P, Nayak S, Rajput VS, Ranjan R, Dayal R. Is there a difference in treatment outcome of tuberculosis patients: Rural Healthcare Providers versus Community Health Workers? J Family Med Prim Care. 2020;9(1):259–63. 10.4103/jfmpc.jfmpc_729_19. PMID: 32110601; PMCID: PMC7014860. PMC7014860 32110601

[10] Anuwatnonthakate A, Limsomboon P, Nateniyom S, Wattanaamornkiat W, Komsakorn S, Moolphate S, Chiengsorn N, Kaewsa-Ard S, Sombat P, Siangphoe U, Mock PA, Varma JK. Directly observed therapy and improved tuberculosis treatment outcomes in Thailand. PLoS ONE. 2008;3(8):e3089. 10.1371/journal.pone.0003089. PMID: 18769479; PMCID: PMC2518105. PMC2518105 18769479

[11] Khachadourian V, Truzyan N, Harutyunyan A, Petrosyan V, Davtyan H, Davtyan K, van den Boom M, Thompson ME. People-centred care versus clinic-based DOT for continuation phase TB treatment in Armenia: a cluster randomized trial. BMC Pulm Med. 2020;20(1):105. 10.1186/s12890-020-1141-y. PMID: 32334553; PMCID: PMC7183136. PMC7183136 32334553

[12] Dye C, Watt CJ, Bleed DM, Hosseini SM, Raviglione MC. Evolution of tuberculosis control and prospects for reducing tuberculosis incidence, prevalence, and deaths globally. JAMA. 2005;293(22):2767-75. 10.1001/jama.293.22.2767. PMID: 15941807. 15941807

[13] Wright J, Walley J, Philip A, Pushpananthan S, Dlamini E, Newell J, Dlamini S. Direct observation of treatment for tuberculosis: a randomized controlled trial of community health workers versus family members. Trop Med Int Health. 2004;9(5):559 – 65. 10.1111/j.1365-3156.2004.01230.x. PMID: 15117299. 15117299

[14] Reis-Santos B, Pellacani-Posses I, Macedo LR, Golub JE, Riley LW, Maciel EL. Directly observed therapy of tuberculosis in Brazil: associated determinants and impact on treatment outcome. Int J Tuberc Lung Dis. 2015;19(10):1188-93. 10.5588/ijtld.14.0776. PMID: 26459531. 26459531

[15] Datiko DG, Yassin MA, Theobald SJ, Blok L, Suvanand S, Creswell J, Cuevas LE. Health extension workers improve tuberculosis case finding and treatment outcome in Ethiopia: a large-scale implementation study. BMJ Glob Health. 2017;2(4):e000390. 10.1136/bmjgh-2017-000390. PMID: 29209537; PMCID: PMC5704104. PMC5704104 29209537

[16] Zhang H, Ehiri J, Yang H, Tang S, Li Y. Impact of Community-Based DOT on Tuberculosis Treatment Outcomes: A Systematic Review and Meta-Analysis. PLoS ONE. 2016;11(2):e0147744. 10.1371/journal.pone.0147744. PMID: 26849656; PMCID: PMC4744041. PMC4744041 26849656

[17] Dam TA, Forse RJ, Tran PMT, Vo LNQ, Codlin AJ, Nguyen LP, Creswell J. What makes community health worker models for tuberculosis active case finding work? A cross-sectional study of TB REACH projects to identify success factors for increasing case notifications. Hum Resour Health. 2022;20(1):25. 10.1186/s12960-022-00708-1. PMID: 35279166; PMCID: PMC8917377. PMC8917377 35279166

[18] Datiko DG, Lindtjørn B. Health extension workers improve tuberculosis case detection and treatment success in southern Ethiopia: a community randomized trial. PLoS ONE. 2009;4(5):e5443. 10.1371/journal.pone.0005443. Epub 2009 May 8. PMID: 19424460; PMCID: PMC2678194. PMC2678194 19424460

[19] Woldesemayat EM, Azeze Z. Treatment outcome of tuberculosis at Dilla Referral Hospital, Gedeo Zone, southern Ethiopia: A retrospective study. PLoS ONE. 2021;16(4):e0249369. 10.1371/journal.pone.0249369. PMID: 33793648; PMCID: PMC8016272. PMC8016272 33793648

[20] Kakame KT, Namuhani N, Kazibwe A, Bongomin F, Baluku JB, Baine SO. Missed opportunities in tuberculosis investigation and associated factors at public health facilities in Uganda. BMC Health Serv Res. 2021;21(1):359. 10.1186/s12913-021-06368-6. PMID: 33865395; PMCID: PMC8052783. PMC8052783 33865395

[21] Suliman Q, Lim PY, Md Said S, Tan KA, Mohd Zulkefli NA. Risk factors for early TB treatment interruption among newly diagnosed patients in Malaysia. Sci Rep. 2022;12(1):745. 10.1038/s41598-021-04742-2. PMID: 35031658; PMCID: PMC8760252. PMC8760252 35031658

[22] Ridolfi F, Peetluk L, Amorim G, Turner M, Figueiredo M, Cordeiro-Santos M, Cavalcante S, Kritski A, Durovni B, Andrade B, Sterling TR, Rolla V. Regional Prospective Observational Research in Tuberculosis (RePORT)–Brazil Consortium. Tuberculosis Treatment Outcomes in Brazil: Different Predictors for Each Type of Unsuccessful Outcome. Clin Infect Dis. 2023;76(3):e930–7. 10.1093/cid/ciac541. PMID: 35788646; PMCID: PMC10169436. PMC10169436 35788646

[23] Matthew Adebayo A, Olaiya Adeniyi B, Oluwasanu M, Hassan A, Ada Ajuwon G, Chidinma Ogbuji Q, Adewole D, John Osho A, Olukolade R, Alabi Ladipo O, Johnson Ajuwon A. Tuberculosis treatment outcomes and associated factors in two states in Nigeria. Trop Med Int Health. 2020;25(10):1261–70. 10.1111/tmi.13467. Epub 2020 Aug 10. PMID: 32677754. 32677754

[24] Katana GG, Ngari M, Maina T, Sanga D, Abdullahi OA. Tuberculosis poor treatment outcomes and its determinants in Kilifi County, Kenya: a retrospective cohort study from 2012 to 2019. Arch Public Health. 2022;80(1):48. 10.1186/s13690-022-00807-4. PMID: 35123570; PMCID: PMC8818215. PMC8818215 35123570

[25] Batista FAM, Araújo JIF, Soares FC, Schlosser TCM, Seixas CT, Nobre TTX. Factors associated with pulmonary tuberculosis in elderly individuals: A protocol for a scoping review. PLoS ONE. 2025;20(2):e0318375. 10.1371/journal.pone.0318375. PMID: 39946387; PMCID: PMC11824960. PMC11824960 39946387

[26] Ryuk DK, Pelissari DM, Alves K, Oliveira PB, Castro MC, Cohen T, Sanchez M, Menzies NA. Predictors of unsuccessful tuberculosis treatment outcomes in Brazil: an analysis of 259,484 patient records. BMC Infect Dis. 2024;24(1):531. 10.1186/s12879-024-09417-7. PMID: 38802744; PMCID: PMC11129366. PMC11129366 38802744

[27] Alsehali A, Alrajih H, Al-Jahdali H, Al-Safi E, Layqah L, Baharoon S, Clinical. Radiological Features and Treatment Outcomes of Tuberculosis in Patients Aged 75 Years and Older. J Epidemiol Glob Health. 2024;14(4):1591–601. 10.1007/s44197-024-00311-8. Epub 2024 Oct 29. PMID: 39470976; PMCID: PMC11652542. PMC11652542 39470976

[28] Tweya H, Feldacker C, Phiri S, Ben-Smith A, Fenner L, Jahn A, Kalulu M, Weigel R, Kamba C, Banda R, Egger M, Keiser O. Comparison of treatment outcomes of new smear-positive pulmonary tuberculosis patients by HIV and antiretroviral status in a TB/HIV clinic, Malawi. PLoS ONE. 2013;8(2):e56248. 10.1371/journal.pone.0056248. Epub 2013 Feb 15. PMID: 23457534; PMCID: PMC3574145. PMC3574145 23457534

[29] Nanzaluka FH, Chibuye S, Kasapo CC, Langa N, Nyimbili S, Moonga G, Kapata N, Kumar R, Chongwe G. Factors associated with unfavourable tuberculosis treatment outcomes in Lusaka, Zambia, 2015: a secondary analysis of routine surveillance data. Pan Afr Med J. 2019;32:159. 10.11604/pamj.2019.32.159.18472. PMID: 31308862; PMCID: PMC6609856. PMC6609856 31308862

[30] Rodgerd A, Morasert T. Effectiveness of Systematic Screening and Treatment of Tuberculosis in Prison in Thailand. Am J Trop Med Hyg. 2024;111(5):1041–5. 10.4269/ajtmh.24-0232. PMID: 39255786; PMCID: PMC11542526. PMC11542526 39255786

[31] Zenbaba D, Bonsa M, Sahiledengle B. Trends of unsuccessful treatment outcomes and associated factors among tuberculosis patients in public hospitals of Bale Zone, Southeast Ethiopia: A 5-year retrospective study. Heliyon. 2021;7(9):e07982. PMID: 34568602; PMCID: PMC8449177. 10.1016/j.heliyon.2021.e07982 PMC8449177 34568602

[32] Hayibor KM, Bandoh DA, Asante-Poku A, Kenu E. Predictors of Adverse TB Treatment Outcome among TB/HIV Patients Compared with Non-HIV Patients in the Greater Accra Regional Hospital from 2008 to 2016. Tuberc Res Treat. 2020;2020:1097581. 10.1155/2020/1097581. PMID: 32832153; PMCID: PMC7424490. PMC7424490 32832153

[33] Woldesemayat EM, Gari T. Tuberculosis treatment outcome of TB/HIV co-infected patients at Adare Hospital, Hawassa City Administration, Sidama Region. BMC Infect Dis. 2024;24(1):1281. 10.1186/s12879-024-10181-x. PMID: 39528948; PMCID: PMC11556195. PMC11556195 39528948

[34] Ockenga J, Fuhse K, Chatterjee S, Malykh R, Rippin H, Pirlich M, Yedilbayev A, Wickramasinghe K, Barazzoni R. Tuberculosis and malnutrition: The European perspective. Clin Nutr. 2023;42(4):486–92. Epub 2023 Feb 10. PMID: 36857957. 10.1016/j.clnu.2023.01.016 36857957

[35] World Health Organization. Guideline: nutritional care and support for patients with tuberculosis. Geneva: World Health Organization. 2013. Available from: https://www.who.int/publications/i/item/9789241506410. Accessed on 24 May 2026. 24624480

[36] Dzhangaziev B, Kulzhabaeva A, Truzyan N, Zhoroev A, Otorbaeva D, Temirbekov S, Shahumyan E, Davtyan K, Isakov T. New approach for tuberculosis contact tracing implemented in the two regions of Kyrgyz Republic during 2017–2018. J Infect Dev Ctries. 2020;14(11.1):109S-115S. 10.3855/jidc.11978. PMID: 33226968. 33226968

[37] Birhane M, Mekonnen S, Dingeta T, Teklemariam Z. Loss to follow-up tuberculosis treatment and associated factors among adults attending at public health facilities in Warder District, Somali Regional State, Eastern Ethiopia. Front Public Health. 2023;11:1151077. 10.3389/fpubh.2023.1151077. PMID: 37234759; PMCID: PMC10208408. PMC10208408 37234759

[38] Lima LV, Pavinati G, Palmieri IGS, Vieira JP, Blasque JC, Higarashi IH, Fernandes CAM, Magnabosco GT. Factors associated with loss to follow-up in tuberculosis treatment in Brazil: a retrospective cohort study. Rev Gaucha Enferm. 2023;44:e20230077. English, Portuguese. 10.1590/1983-1447.2023.20230077.en. PMID: 38055461. 38055461

[39] Tetart M, Meybeck A, Assaf A, Valette M, Choisy P, Blondiaux N, Senneville E. Factors of loss to follow-up during tuberculosis treatment in a low-incidence region. Med Mal Infect. 2020;50(1):28–35. Epub 2019 Mar 16. PMID: 30890281. 10.1016/j.medmal.2019.02.007 30890281

[40] Pavinati G, Lima LV, Teixeira CSS, Hino P, Bertolozzi MR, Nery JS, Magnabosco GT. Vulnerability to loss of follow-up and death due to tuberculosis among homeless individuals in Brazil: a retrospective cohort study. Cien Saude Colet. 2024;29(7):e02742024. 10.1590/1413-81232024297.02742024. Portuguese, English. 38958313

[41] Ngari MM, Mberia JK, Kanana E, Sanga D, Ngari MK, Nyagah DN, Abdullahi OA. Mortality and loss to follow-up among Tuberculosis patients on treatment in Meru County, Kenya: a retrospective cohort study. PLOS Glob Public Health. 2025;5(3):e0003896. 10.1371/journal.pgph.0003896. PMID: 40063632; PMCID: PMC11892847. PMC11892847 40063632

[42] Mando TC, Sandy C, Chadambuka A, Gombe NT, Juru TP, Shambira G, Umeokonkwo CD, Tshimanga M. Tuberculosis cohort analysis in Zimbabwe: The need to strengthen patient follow-up throughout the tuberculosis care cascade. PLoS ONE. 2023;18(11):e0293867. 10.1371/journal.pone.0293867. PMID: 37939099; PMCID: PMC10631662. PMC10631662 37939099

[43] Sharani ZZ, Ismail N, Yasin SM, Zakaria Y, Razali A, Demong NAR, Mohammad M, Ismail Z. Characteristics and determinants of loss to follow-up among tuberculosis (TB) patients who smoke in an industrial state of Malaysia: a registry-based study of the years 2013–2017. BMC Public Health. 2022;22(1):638. 10.1186/s12889-022-13020-3. PMID: 35365112; PMCID: PMC8976383. PMC8976383 35365112

[44] Mulaku MN, Nyagol B, Owino EJ, Ochodo E, Young T, Steingart KR. Factors contributing to pre-treatment loss to follow-up in adults with pulmonary tuberculosis: a qualitative evidence synthesis of patient and healthcare worker perspectives. Glob Health Action. 2023;16(1):2148355. PMID: 36548521; PMCID: PMC9788701. 10.1080/16549716.2022.2148355 PMC9788701 36548521

