# Supplementary material for: Determinants of unsuccessful tuberculosis treatment outcomes among patients under facility-based directly observed therapy (DOT) follow-up in Rwanda (2021–2023): a retrospective analysis of national surveillance data
Source: BMC Infect Dis. 2026 Jul 31;26:1894. doi: 10.1186/s12879-026-14148-y (PMC13715203; doi:10.1186/s12879-026-14148-y)
Supplement: Supplementary file 1 [file 12879_2026_14148_MOESM1_ESM.docx]

**Supplementary File 1:
Bivariate analysis of factors associated with unsuccessful TB treatment outcomes among TB patients under health facility-based DOT in Rwanda**

| **Variable** | **Successful outcome n (%)** | **Unsuccessful outcome n (%)** | **Crude OR** | **95% CI** |
| --- | --- | --- | --- | --- |
| **Age group (years)** |  |  |  |  |
| <40 | 4,654 (57) | 412 (42) | 1.00 | Reference |
| 40–59 | 2,565 (31) | 368 (38) | 1.62 | 1.40–1.88 |
| 60+ | 951 (12) | 199 (20) | 2.36 | 1.97–2.84 |
| **Sex** |  |  |  |  |
| Male | 6,571 (80) | 704 (72) | 1.00 | Reference |
| Female | 1,597 (20) | 274 (28) | 1.60 | 1.38–1.86 |
| **Province of residence** |  |  |  |  |
| Western | 849 (10) | 93 (10) | 1.00 | Reference |
| Northern | 407 (5) | 91 (9) | 2.04 | 1.49–2.79 |
| Kigali City | 2,635 (32) | 421 (43) | 1.46 | 1.15–1.85 |
| Southern | 1,088 (13) | 173 (18) | 1.45 | 1.11–1.90 |
| Eastern | 3,191 (39) | 201 (21) | 0.59 | 0.44–0.74 |
| **Type of treating health facility** |  |  |  |  |
| Correctional Facilities (Prisons) | 3,090 (38) | 71 (7) | 1.00 | Reference |
| Hospitals | 3,210 (39) | 694 (71) | 9.71 | 7.57–12.45 |
| Health Centres / Private Clinics | 1,870 (23) | 214 (22) | 5.05 | 3.84–6.65 |
| **Health facility management** |  |  |  |  |
| Public | 7,244 (89) | 858 (88) | 1.00 | Reference |
| Faith-based & Private | 926 (11) | 121 (12) | 1.10 | 0.90–1.35 |
| **Level of care** |  |  |  |  |
| CT (Centre de Traitement) | 781 (10) | 71 (7) | 1.00 | Reference |
| CDT (Centre de Dépistage et de Traitement) | 7,389 (90) | 908 (93) | 1.35 | 1.05–1.74 |
| **HIV status** |  |  |  |  |
| Negative | 7,191 (88) | 666 (68) | 1.00 | Reference |
| Positive | 976 (12) | 312 (32) | 3.45 | 2.97–4.01 |
| **Currently on cotrimoxazole ^a^** |  |  |  |  |
| No | 471 (49) | 139 (45) | 1.00 | Reference |
| Yes | 494 (51) | 169 (55) | 1.16 | 0.90–1.50 |
| **TB contact history** |  |  |  |  |
| Yes | 1,455 (18) | 46 (5) | 1.00 | Reference |
| No | 6,715 (82) | 933 (95) | 4.39 | 3.25–5.94 |
| **BMI at treatment initiation** |  |  |  |  |
| ≥18.5 kg/m² | 1,027 (13) | 112 (11) | 1.00 | Reference |
| <18.5 kg/m² | 7,143 (87) | 867 (89) | 1.11 | 0.90–1.37 |
| **Receipt of TB nutritional support** |  |  |  |  |
| Yes | 1,887 (23) | 216 (22) | 1.00 | Reference |
| No | 6,283 (77) | 763 (78) | 1.06 | 0.90–1.24 |
| **Site of TB disease** |  |  |  |  |
| Pulmonary TB | 6,107 (75) | 720 (74) | 1.00 | Reference |
| Extra-pulmonary TB | 2,063 (25) | 259 (26) | 1.06 | 0.92–1.24 |
| **Method of TB confirmation** |  |  |  |  |
| Bacteriologically confirmed | 5,314 (65) | 563 (58) | 1.00 | Reference |
| Clinically diagnosed | 2,856 (35) | 416 (42) | 1.37 | 1.20–1.57 |

N = 9,149 total patients: OR = Odds Ratio; CI = Confidence Interval; CT = Centre de Traitement; CDT = Centre de Dépistage et de Traitement; BMI = Body Mass Index; TB = Tuberculosis ^a^Cotrimoxazole analysis restricted to HIV-positive patients only (n = 1,273)
